# Severe pulmonary edema after pulmonary hypertension interventional surgery: case report and literature review

**DOI:** 10.3389/fmed.2025.1723147

**Published:** 2026-01-12

**Authors:** Tuo Shen, Xingping Lv, Chun Wang, Qimin Ma, Ruihua Wang, Shaolin Ma, Feng Zhu

**Affiliations:** Department of Critical Care Medicine, Shanghai East Hospital, School of Medicine, Tongji University, Shanghai, China

**Keywords:** case report, fibrosing mediastinitis, interventional therapy, pulmonary edema, pulmonary hypertension, right heart catheterization

## Abstract

**Background:**

Pulmonary hypertension caused by fibrosing mediastinitis often presents with clinical manifestations related to involvement of the pulmonary arteries, pulmonary veins, and bronchi. Interventional therapy has become an important treatment option; however, it carries a significant risk of complications. In this case, the patient developed severe pulmonary edema after intervention, which posed major challenges to clinical management.

**Case presentation:**

A middle-aged man presented with recurrent cough, expectoration, chest tightness, and dyspnea. He was initially treated for chronic obstructive pulmonary disease, but his symptoms showed little improvement. Owing to severe pulmonary hypertension, he was referred to the Department of Respiratory Medicine, where right heart catheterization confirmed the diagnosis of fibrosing mediastinitis. He subsequently underwent balloon angioplasty of the left pulmonary artery. During a second admission, he received right pulmonary artery balloon angioplasty with stent implantation under right heart catheter guidance. Shortly after the procedure, he developed severe pulmonary edema complicated by shock and acute renal failure. Conservative treatment was unsuccessful, and veno-venous extracorporeal membrane oxygenation (VV-ECMO) was initiated. With ECMO support, circulation was stabilized through active fluid resuscitation, followed by careful adjustment of volume status. His pulmonary edema gradually resolved, and he was eventually discharged in full recovery.

**Conclusion:**

Pulmonary hypertension caused by fibrosing mediastinitis is associated with poor prognosis. Interventional therapy may improve symptoms and hemodynamics but carries the risk of complications such as pulmonary edema, vascular injury, restenosis, and in-stent thrombosis. In this case, the development of severe pulmonary edema was most likely related to insufficient evaluation of the pulmonary venous system and limitations in the diagnostic and therapeutic process. This emphasizes the importance of comprehensive assessment of pulmonary venous involvement before intervention.

## 1 Introduction

Pulmonary hypertension (PH) is a heterogeneous clinical syndrome characterized by structural or functional alterations of the pulmonary vasculature, resulting in increased pulmonary vascular resistance and pulmonary arterial pressure. Without timely intervention, PH inevitably progresses to right heart failure and death (1). Pulmonary hypertension caused by fibrosing mediastinitis (PH-FM) is classified as Group 5 in the WHO PH classification. Although FM is a benign disease, the absence of effective therapeutic options results in a 5-year mortality rate of up to 46% (2).

The etiologies of FM include infectious agents, sarcoidosis, autoimmune diseases, iatrogenic injury, and idiopathic causes. Clinical presentation varies depending on the anatomical extent of fibrotic infiltration (3). PH-FM can be divided into three subtypes: (1) involvement of the pulmonary arteries (PA) and adjacent bronchi without pulmonary vein (PV) obstruction; (2) isolated PV involvement with sparing of the PA and bronchi; and (3) simultaneous involvement of the PA, PV, and bronchi (4). In China, *Mycobacterium tuberculosis* is the predominant cause of PH-FM, with most cases presenting as the third subtype (3, 5). Diagnosis relies primarily on computed tomography (CT) and right heart catheterization (6), while chest radiography may reveal characteristic “two-sign” or “three-sign” patterns (4). Interventional therapy is generally regarded as the first-line treatment for PH-FM (7–9). Importantly, PV intervention should be prioritized, whereas PA intervention should be delayed until restoration of PV patency to avoid hemodynamic imbalance and subsequent complications (4).

Here, we report the case of a middle-aged man with PH-FM who developed severe pulmonary edema following right heart catheter-guided interventional therapy. Although he ultimately recovered after intensive management, this case highlights the importance of comprehensive evaluation of the pulmonary vasculature and careful formulation of individualized treatment strategies to minimize the risk of life-threatening complications.

## 2 Case presentation

### 2.1 Patient information

A 60-year-old male was admitted to the hospital with a 10-year history of recurrent cough, expectoration, chest tightness, and shortness of breath. He reported no chest pain, hemoptysis, or other associated symptoms. He had previously received treatment for “chronic obstructive pulmonary disease (COPD)” at another hospital, but with poor therapeutic response. An echocardiography performed at the prior hospital indicated severe PH. The patient had no significant family history of genetic diseases, and his history of tuberculosis was unclear.

### 2.2 Clinical findings

On physical examination, vital signs were within the normal range. Bilateral lung auscultation revealed coarse breath sounds, with localized moist rales audible in the right lung field. A loud second heart sound (P_2_) was noted. There were no signs of systemic venous congestion or limb edema. No other significant abnormalities were found on the remaining physical examination.

### 2.3 Diagnostic assessment

**Figure 1:**
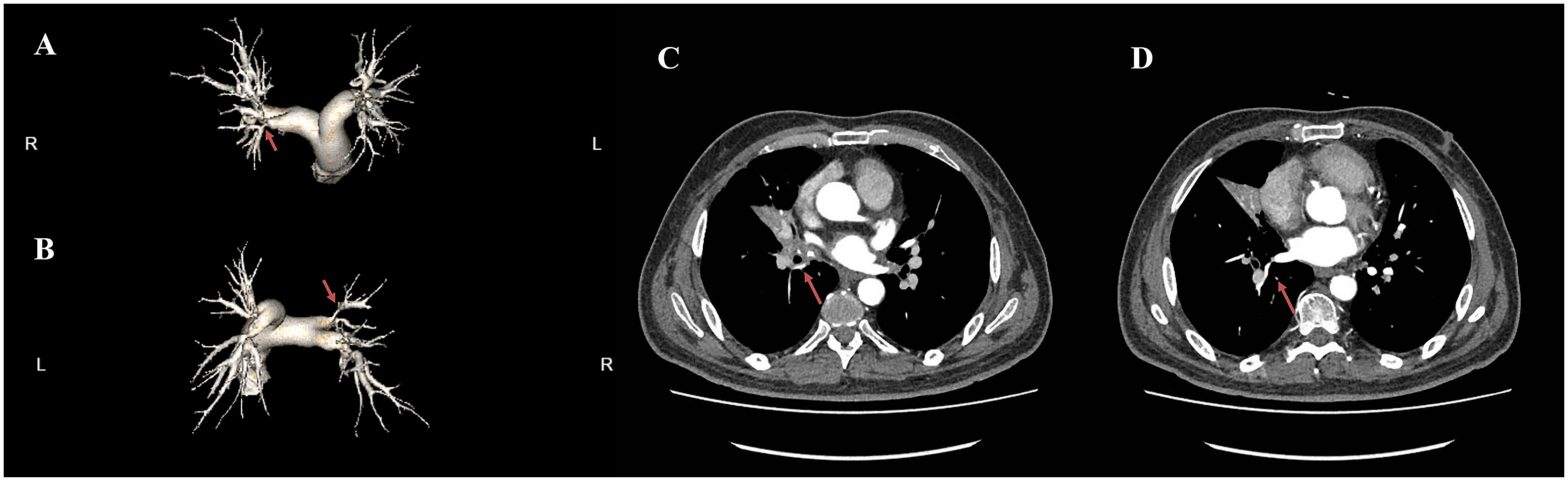
CTPA. **(A,B)** Three-dimensional reconstruction shows stenosis at the bilateral PA branches (indicated by red arrows). **(C,D)** Axial view (venous phase) suggests suspected stenosis at the branches of the right middle and right lower PV.

**Figure 2:**
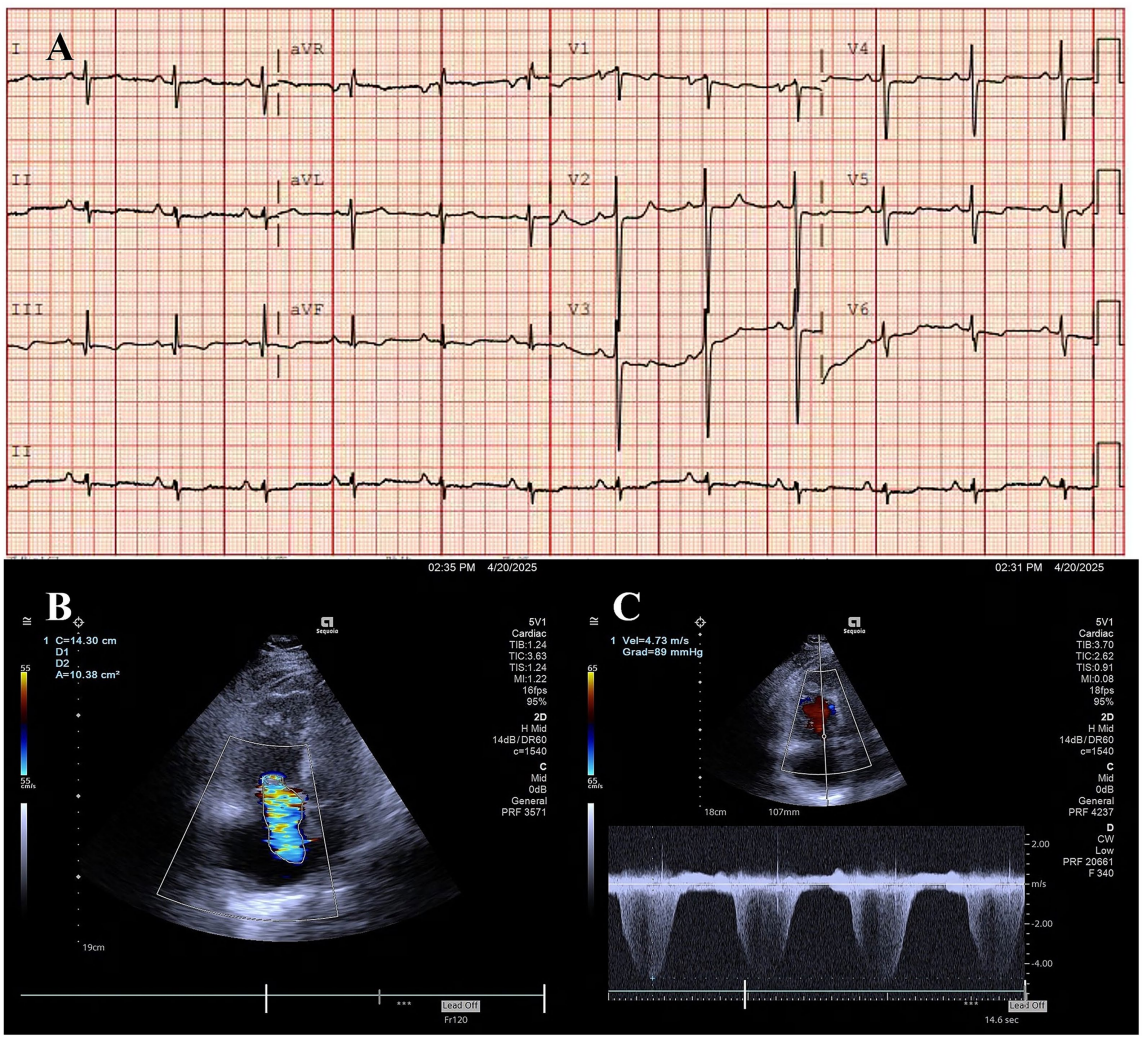
ECG and echocardiography. **(A)** ECG findings are consistent with PH. **(B,C)** Echocardiography shows severe tricuspid regurgitation, with a peak velocity of tricuspid regurgitation of 4.73 m/s.

**Figure 3:**
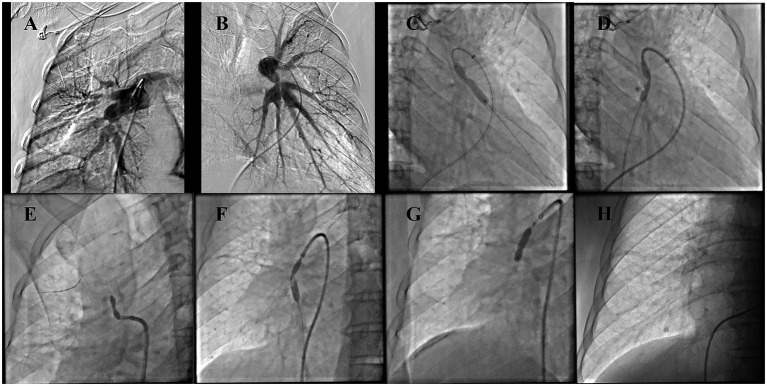
Right heart catheterization. **(A,B)** Multiple stenoses in the branches of the bilateral PA. **(C,D)** Balloon angioplasty performed on LA8 and LA10. **(E,F)** Balloon angioplasty performed on RA3 and RA8. **(G,H)** Balloon angioplasty with stent implantation performed on PA9.

Computed tomography pulmonary angiography (CTPA) showed: multiple irregular soft tissue density lesions in the mediastinum and bilateral hilar regions, atelectasis of the right middle lobe, tortuosity of the proximal segments of bilateral PA branches with localized external compressive stenosis, and dilatation of the main PA and its left/right branches (Figures 1A–D). Electrocardiography (ECG) demonstrated clockwise rotation, with ST-segment depression and biphasic or inverted T-waves in leads II, III, aVF, and V2–V6 (Figure 2A). Echocardiography revealed: enlargement of the right atrium and right ventricle, moderate-to-severe tricuspid regurgitation, and a systolic pulmonary arterial pressure (sPAP) of 104 mmHg (Figures 2B,C). Right heart catheterization showed: annular stenosis at the ostia of right pulmonary artery (RA) segments 1–5 and 8–10, annular stenosis at the ostia of left pulmonary artery (LA) segments 3 and 8–10, a pulmonary vascular resistance (PVR) of 26.23 Wood units (WU), and a pulmonary capillary wedge pressure (PCWP) of 8 mmHg (Figures 3A,B).

### 2.4 Therapeutic intervention

The patient underwent two successive right heart catheter-guided interventional procedures. First, he received balloon angioplasty for the LA8 and LA10. Postoperatively, his symptoms improved subjectively with no significant complications; a follow-up echocardiogram showed a 10 mmHg reduction in sPAP.

One month later, the patient underwent a second intervention, which included balloon angioplasty for the RA3 and RA8 and stent implantation with balloon angioplasty for the RA9 (Figures 3C–H). Two hours post-procedure, he developed chest tightness, shortness of breath, and profuse sweating, without hemoptysis or chest pain. Despite high-flow oxygen therapy [50 L/min, 80% fraction of inspired oxygen (FiO₂)], his peripheral oxygen saturation (SpO₂) remained at 88%. Endotracheal intubation and mechanical ventilation were initiated. Laboratory testing revealed a D-dimer level of 1.7 mg/L. Flexible bronchoscopy showed no obvious airway foreign bodies or obstruction, but noted white frothy exudate; auscultation revealed bilateral pulmonary moist rales, which were more prominent on the right. Reperfusion pulmonary edema was suspected.

**Figure 4:**
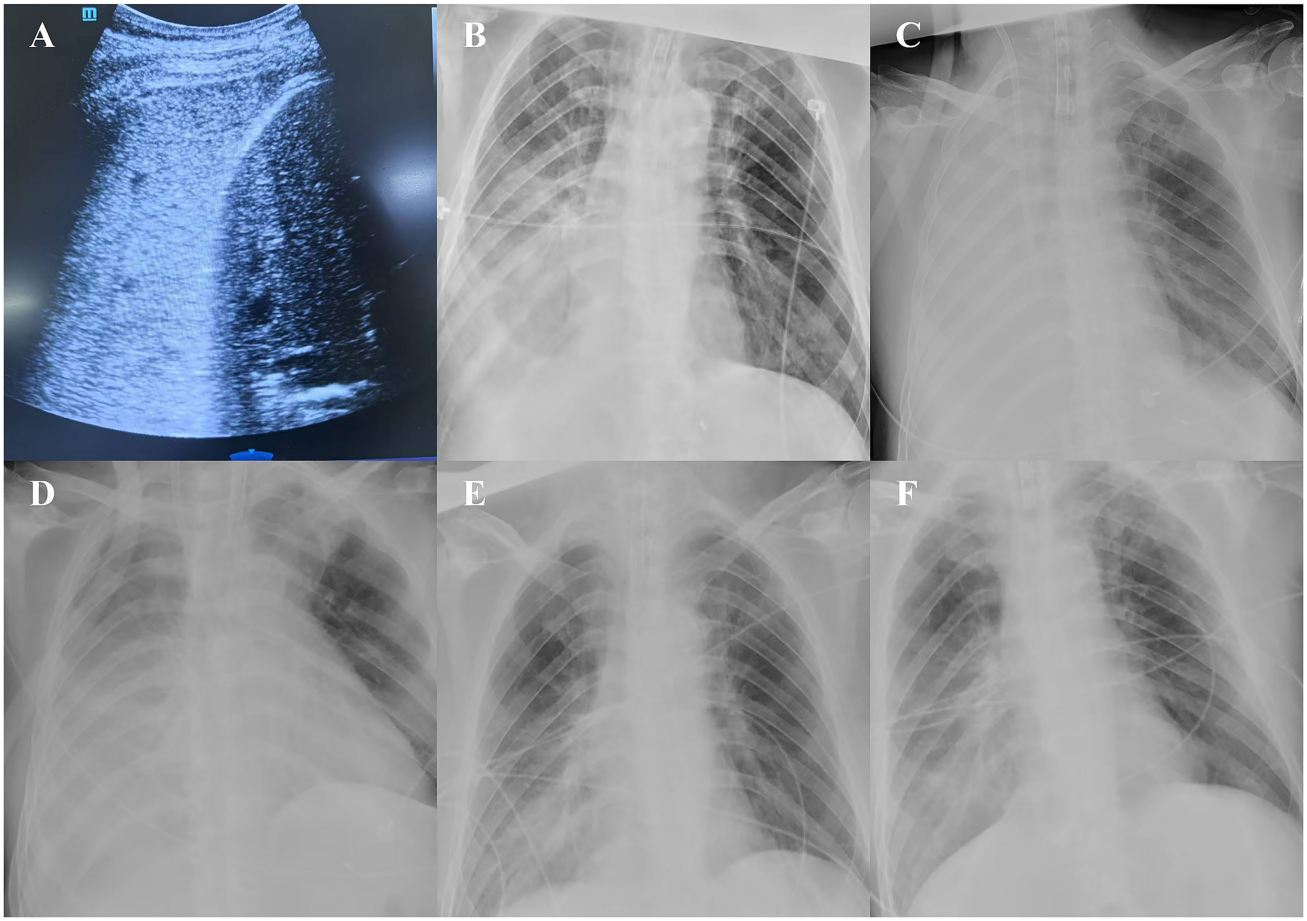
Ultrasonography and chest radiography. **(A)** Chest ultrasonography shows “hepatic-like consolidation” in the right lower lung. **(B)** Chest radiograph corresponding to the ultrasonographic findings (before ECMO support and fluid resuscitation). **(C)** Chest radiograph after fluid resuscitation with ECMO support. **(D–F)** Chest radiographs during the resolution phase.

After initiating lung-protective ventilation [tidal volume: 6 mL/kg, positive end-expiratory pressure (PEEP): 12 cmH₂O], the patient’s oxygenation index (P/F) did not improve (P/F <60 mmHg), and he developed severe hypoperfusion and shock: heart rate was 140 beats per minute, and high-dose vasoactive agents (norepinephrine at 1 μg/kg/min) were required to maintain a mean arterial pressure >60 mmHg; anuria was also present. Bedside ultrasound showed right lower lung consolidation (Figure 4A), an inferior vena cava diameter <5 mm, and significantly reduced left ventricular end-diastolic volume, with preserved biventricular systolic function. Findings on bedside chest radiography were consistent with the ultrasound and physical examination results (Figure 4B).

Reviewing the medical history, the patient received diuretic therapy during this hospitalization with a negative fluid balance of approximately 1,000 mL per day. At this time, there was a contradiction between the treatment of hypoxemia caused by severe pulmonary edema and that of hypovolemia. Therefore, right femoral vein-right internal jugular vein veno-venous extracorporeal membrane oxygenation (VV-ECMO) support was initiated.

In the early stage of ECMO operation, the blood flow could only be maintained at 1 L/min, and frequent pipeline shaking occurred. Crystalloid fluid resuscitation was then administered at a rate of 10 mL/kg/h. Under the dynamic guidance of bedside ultrasonography [assessing left ventricular end-diastolic volume, inferior vena cava (IVC) diameter and its variability], after a cumulative infusion of approximately 3,000 mL of crystalloid fluid, the patient’s hemodynamics gradually stabilized, the demand for vasoactive drugs decreased significantly (norepinephrine at 0.1 μg/kg/min), and urine output recovered. The ECMO blood flow could be maintained at 2–3 L/min, and the patient’s peripheral capillary oxygen saturation (SpO₂) remained between 92 and 98%.

Lung-protective ventilation was continued, and volume status was optimized; his P/F gradually improved, and chest radiography showed progressive resolution of right pulmonary exudation and consolidation (Figures 4D–F). The patient was weaned off ECMO on post-ECMO day 5 and off mechanical ventilation on day 10, and was discharged successfully after 3 weeks with full recovery. During the subsequent 2-month follow-up, the patient remained in good recovery. However, due to this medical experience, the patient expressed concerns about whether to undergo interventional treatment again.

## 3 Discussion

In FM-PH, electrocardiographic and echocardiographic findings primarily reflect right heart involvement secondary to PH, with no pathognomonic features. While chest radiography has limited diagnostic value for FM-PH, it often reveals a “two-sign pattern” (prominent PA, atelectasis) or “three-sign pattern” (additionally with pleural effusion, often indicative of PV involvement). These patterns facilitate the early identification of FM-PH in clinical practice (4).

In the present case, no typical two-sign or three-sign pattern was observed on chest radiography, which may be attributed to the location and extent of mediastinal involvement. The presence of right middle lobe atelectasis was confirmed by CT. CTPA and computed tomography pulmonary venography (CTPV) are widely regarded as key diagnostic tools for FM-PH (10). In this patient, CTPA demonstrated multiple irregular soft tissue infiltrates in the mediastinum and bilateral hilar regions, right middle lobe atelectasis, tortuosity of the proximal segments of bilateral PA branches, and localized external compressive stenosis (Figures 1C,D). Further right heart catheterization—considered the gold standard for clinical diagnosis of FM-PH (10)—confirmed PA stenosis and ruled out intravascular thromboembolism. Based on these findings, a clinical diagnosis of FM-PH was reasonable. Unfortunately, no further histopathological examination was performed, likely due to clinicians’ concerns about potential complications associated with invasive pathological sampling. In contrast to conventional surgical biopsy, You et al. (11) recently explored endobronchial mediastinal cryobiopsy, a procedure that enables the acquisition of necessary mediastinal tissue while reducing the risk of complications.

Current treatment options for FM-PH include conservative management, surgical intervention, and interventional therapy. However, their efficacy remains limited, and controversies persist. Conservative management primarily focuses on symptomatic treatment for PH and heart failure. Glucocorticoid efficacy varies by FM subtype (granulomatous vs. non-granulomatous) (4), highlighting the value of definitive pathological results for guiding treatment. Surgical treatment—targeting pulmonary vascular, airway, or mediastinal involvement—is technically challenging, with a surgical mortality rate of up to 20%; additionally, 42% of patients experience recurrence postoperatively, requiring additional surgery or alternative therapies (7, 12). Thus, surgical treatment is currently selected with caution. Interventional therapy is a promising yet controversial option, primarily due to its associated complications and the challenges in formulating treatment strategies based on the varying patterns of pulmonary vascular involvement (8). As previously noted, interventional therapy should prioritize PV assessment: addressing PA stenosis without relieving PV stenosis may lead to severe pulmonary hemodynamic mismatch and subsequent complications (13).

Severe pulmonary edema occurred during treatment in this case, potentially due to over-aggressive intervention for PA stenosis and inadequate assessment of PV stenosis. No obvious pleural effusion was observed at admission, and right heart catheterization revealed a normal PCWP (8 mmHg), suggesting the absence of severe PV stenosis. However, this did not completely rule out localized PV stenosis. Retrospective review of the initial CTPA (venous phase) indicated possible stenosis of the right middle and lower PV. In this context, after stent placement significantly improved local blood flow, increased venous return resistance led to severe pulmonary edema. This underscores that while endovascular interventional therapy is invasive, it can effectively alleviate pulmonary vascular stenosis; however, it may still cause severe complications if it results in pulmonary arteriovenous hemodynamic mismatch. Additionally, complications such as post-interventional vascular restenosis and in-stent occlusion require attention and further resolution. The patient’s long-term outcome will be monitored in subsequent follow-ups.

The pulmonary edema in this case was severe, accompanied by both severe respiratory failure and shock. Clinically, pulmonary edema is typically managed with positive-pressure ventilation and diuresis; however, this patient also had volume depletion requiring fluid resuscitation, creating a significant clinical dilemma. Given the absence of residual oxygenation reserve, fluid resuscitation was administered under VV-ECMO support—despite the potential for improving hypoxemia secondary to low cardiac output from volume depletion. While this decision may be controversial, the patient ultimately benefited clinically. Subsequent chest radiography confirmed that pulmonary edema worsened following further fluid resuscitation (Figure 4C). Although VV-ECMO is not a standard treatment for post-interventional pulmonary edema in FM-PH, it provides an alternative option for managing extreme clinical cases.

## 4 Conclusion

The management of FM-PH poses significant clinical challenges. Interventional therapy, as a potentially effective treatment modality, has garnered increasing attention. While this case highlights severe pulmonary complications that may occur post-intervention, it is not intended to discourage the use of interventional therapy. Instead, it emphasizes that clinicians must conduct a comprehensive assessment of the pulmonary vasculature before initiating interventional therapy to develop safe and effective treatment strategies. Pulmonary veins should be assessed prior to any intervention on pulmonary arteries, and the treatment of pulmonary artery stenosis should be carried out in a gradual and stepwise manner. Particularly when the assessment of pulmonary veins is insufficient, simultaneous intervention on multiple sites of pulmonary artery stenosis should be avoided.

## Data Availability

The original contributions presented in the study are included in the article/Supplementary material, further inquiries can be directed to the corresponding author.
