# Supplementary material for: Severe pulmonary edema after pulmonary hypertension interventional surgery: case report and literature review
Source: Front Med (Lausanne). 2026 Jan 12;12:1723147. doi: 10.3389/fmed.2025.1723147 (PMC12833460; doi:10.3389/fmed.2025.1723147)
Supplement: Supplementary file 1 [file Table_1.DOCX]

## Table showing the timeline of cares

| **Date** | **Event** | **Outcome** |
| --- | --- | --- |
| Apr. 2025 | Recurrent cough, expectoration, chest tightness, and shortness of breath (10 years) | Initial presentation; diagnostic workup initiated. |
|  | Diagnostic testing | Pulmonary hypertension (Echocardiography).  Atelectasis; Localized external compressive stenosis of PA(CTPA).  External compressive stenosis of both PA, no thrombus detected, suspected FM-PH. (Right heart catheterization) |
| May 2025 | Right heart catheterization with stent implantation | Developed chest tightness, shortness of breath, and profuse sweating. (2h later) |
|  | Endotracheal intubation and mechanical ventilation | Oxygenation index (P/F) < 60mmHg; Severe hypoperfusion and shock. |
|  | Bedside ultrasound and chest radiography | Right lower lung consolidation. |
|  | VV-ECMO | Ran for 5 days. |
| Jun. 2025 | Fluid resuscitation, lung-protective ventilation strategy, volume optimization | Pulmonary edema resolved gradually. |
|  | ECMO withdrawal | 5 days later. |
|  | Ventilator withdrawal | 10days later. |
|  | Regular follow-up | The patient was discharged from the hospital with full recovery after 3 weeks. |
